# The War Tax

**Published:** 1918-01

**Authors:** 


### The War Tax.

In peace times, the management of our country has cost about $10 per capita per year. The direct and bonded war tax is about $110. What it will be in future years, can scarcely be guessed at. This tax is however, by no means so important as the indirect tax in the form of increased prices. Even before the war, the idea was gradually being accepted that the resources and industries of the country belonged to the whole people, not necessarily equally, not even proportionately to the value of the activities of the individual citizen but with some slight attention to the latter factor and with the dawn of the conception that mere exploitation of the value of one man's services by another or of national wealth by individuals, should be discouraged.

In the long run, it makes little difference, up to a reasonable limit which not even the exigencies of war are likely to exceed, how much one pays or how much the price of any one commodity increases, providing the total budget of the average man or family is not unduly increased. It does not even matter much whether the increase is for "necessities," providing the cost of comforts and certain luxuries can be correspondingly reduced. Doubling the price of bread, or sugar, or meat, is by no means so serious as might at first seem, if we can, some how or other, compensate for it in other ways. Railroad travel or the sending of a telegram may be quite as necessary in particular cases and the availability of telephone connection actually saves an enormous expense and may double or treble the efficiency of a man or a business. It is worth while considering that all three of these utilities cost us considerably more than they do the inhabitants of most other countries. For example, all along the Atlantic coast, where railroading involves few engineering problems, where the installation was complete years ago and requires merely maintenance and gradual improvement, where the volume of travel allows the greatest profit and economy of management, the prevailing rates are almost exactly 50% higher than for most parts of Europe and many parts of our own country. In some respects, the service is slightly better than in Europe but this superiority is not of great practical moment to the average traveler nor does it involve a proportionate increase in expense.

Last year, various oil companies reported profits of 50% on investment. Gasoline sells at over 200% of the formerly established profitable price. There is an increased demand but this, in itself, means greater profit, as the expense of production increases only for certain items of the manipulation. There is a decrease in supply only in the sense that guesses at the total natural supply at present known to exist, allow the estimate that it will last only for about 25 years. This is ample time for the development of substitute fuels or substitute means of power.

Hotels and restaurants which formerly threw in half a cent's worth of bread with orders representing 300-500% gross profit on foods, are now compelled to charge 10 cents for a cent's worth, at the present price. Hotels catering mainly to men of moderate means, not only charge $3 for a night's lodging but announce the charge as reasonable although the lower half of the 10% of the population who have the greatest wealth, spend on the average 10-20 cents for this necessity. The very moderately skilled laborer who brings a meal from the kitchen to the table, or performs some similar menial task, expects something like the highest pay of the mechanic or clerical employee in addition to a salary that may be low but that represents about all his labor is worth.

The people are being urged not to hoard foods, but are not adequately protected against arbitrary and sudden increase in price. They are urged to substitute other grains for wheat but, over the counter, are charged more for the cheaper grains than for the more satisfactory staple of life. All sorts of industries, formerly conducted by individuals, such as washing, supplying milk, making repairs of all kinds, manufacturing articles of a domestic nature, blacking shoes, etc., are being taken over by firms for conduct on economic, wholesale lines, and the rates have been increased by 100% or more.

It is high time for the people to wake up to the fact that the old idea of selling labor and commodities at a fair value is being superseded by that of shearing velvet, that inevitable —and not always inevitable—increases in cost of raw material and labor are being passed on to the ultimate consumer with the addition of double or treble the actual increase in cost. Men of very moderate means—so far as can be judged, considerably less than 10% of the population have incomes of $2,000 and more—are expected to pay for any particular item like millionaires. That this expectation has largely succeeded, is due mainly to the fact that they have themselves pretended to be able to do so.

The war may compel us to look facts squarely in the face, to drop this pretense, and to insist on a scale of valuation based on actual necessary cost and reasonable profit. If so, it will pay for itself. If not, it is difficult to see how we can continue without ruin.

